# Conservative Treatment of Camptodactyly with use of Orthoses: A Retrospective Cohort

**DOI:** 10.1055/s-0044-1786199

**Published:** 2024-09-04

**Authors:** Maria da Conceição Soares de Oliveira, Felipe Soares Figueiredo, Diego Pinheiro Aguiar

**Affiliations:** 1 Divisão de Pesquisa, Área de Reabilitação, Instituto Nacional de Traumatologia e Ortopedia Jamil Haddad (INTO), Rio de Janeiro, RJ, Brasil; 2 Instituto Nacional de Traumatologia e Ortopedia Jamil Haddad (INTO), Rio de Janeiro, RJ, Brasil

**Keywords:** finger joint, hand deformities, congenital, occupational therapy, orthosis, rehabilitation

## Abstract

**Objective** To evaluate the outcomes of conservative treatment using static orthoses manufactured by the Occupational Therapy Sector of our institution in participants with camptodactyly types I, II, and III in their rigid or flexible forms, to describe the demographic and clinical data, and to analyze the number of dropouts during the treatment period.

**Methods** The Ethics in Human Research Committee approved the project under protocol number CAAE 20300419.3.0000.5273. All medical records used in the research were made available by our institution. In the present retrospective study, we did not use the informed consent form due to the impossibility of contacting the high number of participants. The study included medical records of 38 participants treated at the Occupational Therapy Outpatient Clinic from 2013 to 2019.

**Results** Of the 54 fingers treated with orthoses, 38 were completely corrected. The rates of correction were as follows: type I in its rigid form – 100% type I in its flexible form – 81.2%; type II in its rigid form – 0%; type II in its flexible form – 100%; type III in its rigid form – 47.6%; and type III in its flexible form – 100%. Of the 93 fingers included in this study, 42% abandoned the treatment.

**Conclusion** Static orthoses are a safe alternative to surgical procedures, with low execution complexity for camptodactyly treatment.

## Introduction

**Fig. 1:**
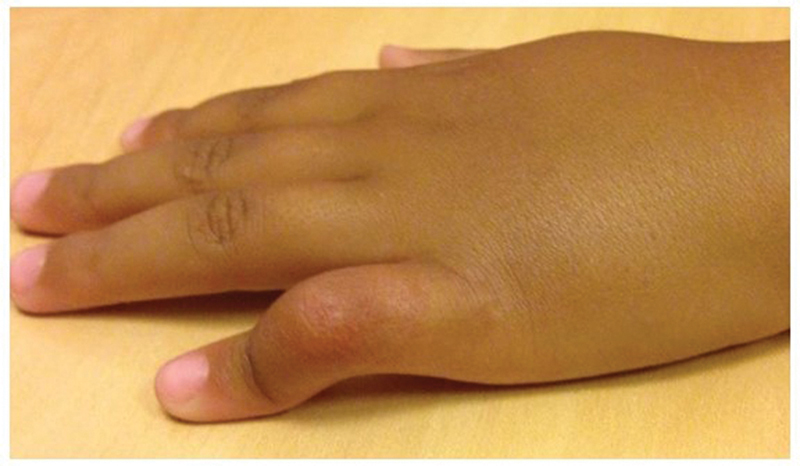
Clinical presentation of a patient with camptodactyly. Flexion of the proximal interphalangeal (PIP) joint of the fifth finger.

Camptodactyly is a permanent flexion deformity of the proximal interphalangeal (PIP) joint in one or more fingers, except the thumb, of non-traumatic cause. 1 The fifth finger is generally the most affected, and it may present hyperextension of the metacarpophalangeal joint (MCP). 2 3 For Monteiro and Almeida, 4 camptodactyly is a flexion deformity in the anteroposterior plane ( Fig. 1 ).

**Fig. 2:**
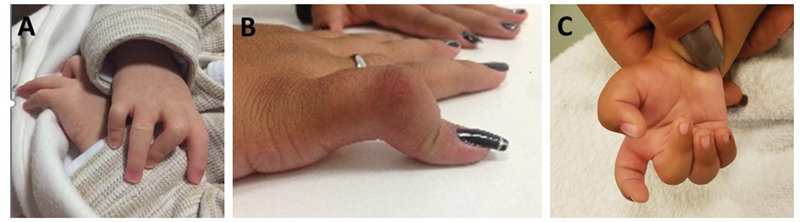
Types of camptodactyly according to the Benson et al. 5 classification. ( **A** ) Type I: PIP joint flexion in the middle and little fingers. ( **B** ) Type II: PIP joint flexion of the little finger. ( **C** ) Type III: PIP joint flexion in the middle, ring, and little fingers.

According to the classification by Benson et al., 5 camptodactyly consists of the following types: Type I – children's camptodactyly, the most common form, which often affects the little finger alone ( Fig. 2A ); type II – adolescent camptodactyly, which predominates in females and presents clinically as type-I ( Fig. 2B ); type III – it is present since birth, affects several fingers, and is often bilateral and associated with syndromes and other malformations; in addition, it features rigid and accentuated shapes ( Fig. 2C ).

Camptodactyly is painless and affects approximately 1% of the population. 6 Some families present an autosomal dominant trait with variable penetrance. 7 In other cases, the deformity appears casually. Approximately 75% of the cases are bilateral and progress during the skeletal growth spurt, mainly in the age groups ranging from 1 to 4 and 10 to 14 years. 4

The fingers of camptodactyly subjects present several abnormal structures. Numerous abnormalities have been implicated as the primary cause, but with no consensus. 8 It can manifest as changes in various structures responsible for joint balance.

The patient may present shortening of the superficial flexor tendon of the finger, of the skin, of the PIP joint capsule, and aponeurosis, in addition to abnormalities in lumbrical muscle attachment, or intrinsic muscle insufficiency. All of these structures have been related to camptodactyly, but none of them has been proven to be an etiological factor. 1 2 8 9 10 11 12 13 14

**Fig. 3:**
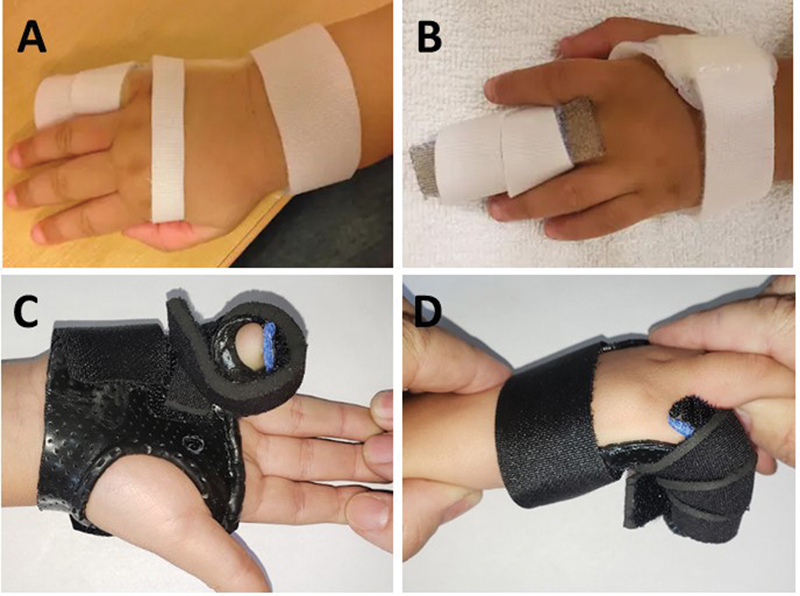
Models of static orthoses with dorsal support used for type-I camptodactyly. ( **A** ) Static orthosis for flexible camptodactyly on the little finger; ( **B** ) static orthosis for flexible camptodactyly in the middle finger; ( **C** ) static orthosis for rigid camptodactyly in the little finger with flexed metacarpophalangeal (MCP) joint, volar view; and ( **D** ) static orthosis for rigid camptodactyly in the little finger with flexed MCP joint, lateral view.

**Fig. 4:**
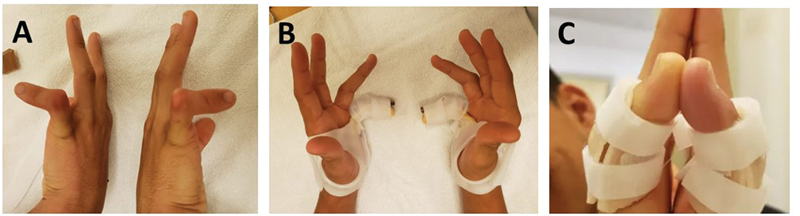
Models of static orthoses with dorsal support for rigid type-II camptodactyly. ( **A** ) Bilateral rigid type-II camptodactyly: PIP joint flexion in the little finger; ( **B** ) static orthosis for rigid camptodactyly in the little finger with flexed MCP joint, dorsal view; and ( **C** ) short static orthoses for daytime use.

**Fig. 5:**
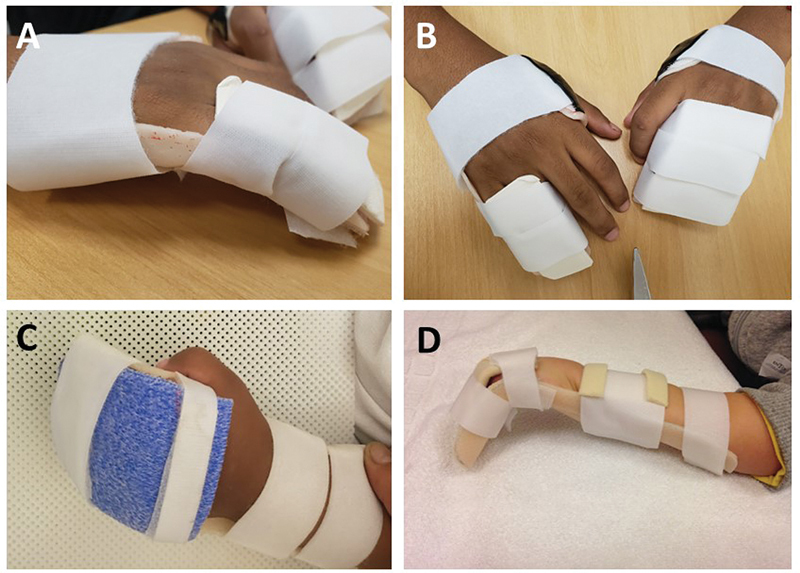
Models of static orthoses with dorsal support used for rigid type-III camptodactyly. ( **A** ) Static orthosis for rigid camptodactyly in the little and ring fingers, lateral view; ( **B** ) static orthoses for rigid camptodactyly in the little and ring fingers of the right hand, and the little, ring and middle fingers of the left hand; ( **C** ) static orthosis for camptodactyly encompassing all fingers, with flexed MCP joint; and ( **D** ) static orthosis for camptodactyly encompassing all fingers and flexed MCP joint.

Goffin et al. 15 reported that hand function is not affected in patients with up to 30° of flexion, with functional compromise above 60°. The most common complication of the surgical treatment for camptodactyly correction is the loss of total flexion of the finger. Almeida et al. 16 stated that incomplete extension is more desirable than deficient flexion. Patients must use orthoses even after the surgical treatment. 17 18 19 The use of orthoses ( Figs. 3A-D , 4A-C , and 5A-D ) for camptodactyly treatment is based on the principle of soft tissue remodeling, a fundamental concept for the theory and technique of immobilization known empirically since ancient times. Slow, gentle, prolonged stress causes soft tissues to remodel or grow. 20

The primary reason to select an orthosis is the biological principle of connective tissue remodeling. This remodeling is a response to time and stress inflicted on the connective tissue, and it consists of a physical rearrangement of the tissue's extracellular matrix. It is not a quick process, and it is directly associated with the frequency of use and total time of daily use. 21 Joint contracture correction requires a constant and low-load force applied for a sufficient period to cause stretching and the creation of new tissue. Adjustments are also necessary to accommodate the changes achieved and maintain the alignment of mechanical force. Despite controversies regarding the mechanism of action on soft tissue, the result is an adaptation to the presence or absence of force, increasing or decreasing in size and remodeling to accommodate the stress received. 22 Living tissues have mechanical sensors to detect pressure, direct force changes, touch, and stretching. The key is to establish the amount of stimulus needed to promote cellular tissue modification and remodeling. Although the first observations of the influence of forces on the shape and function of tissues occurred around 150 years ago, there is still much to uncover. 23

The present study is relevant due to several camptodactyly types and structures potentially involved. In addition, the alternative of surgical treatment often results in the loss of complete flexion in the treated finger.

We aimed to evaluate the conservative treatment with static orthoses for all types of camptodactyly in patients treated at our service.

## Materials and Methods

The Ethics in Human Research Committee approved the project of the present study under protocol number CAAE 20300419.3.0000.5273. All medical records used in the research were made available by our institution. The present retrospective study did not use the informed consent form due to the impossibility of contacting the high number of participants.

### Study Design

The present observational retrospective cohort study included six cohorts, one for each type of camptodactyly (I, II, or III) with rigid or flexible presentation according to the Benson et al. classification. 5 For each of the six cohorts, we evaluated the proportion of patients achieving correction and the average time to correct the type and form of camptodactyly. We also assessed the frequency of affected fingers and the dropout rate from the conservative treatment. We considered camptodactyly correction as complete extension of the finger without loss of flexion.

### Sample Composition

The present study included medical records from participants diagnosed by the Specialized Care Center (SCC) of the Hand with camptodactyly of types I, II, and III in their rigid or flexible forms. All medical records from patients undergoing surgery for other associated deformities or with another type of congenital deformity not related to camptodactyly were excluded.

### Data Collection

We used the Epi Info (Centers for Disease Control and Prevention, Atlanta, GA, United States) software, version 7.2.3.1, and developed a form-type instrument specifically for the present research. Data collection followed the protocol created for the current study. We collected the demographic data of the 38 patients, in addition to camptodactyly data (affected finger, type and form of camptodactyly), as well as data on corrections and dropouts, and treatment time (date of birth, dates of the beginning and end of the follow-up, date when the camptodactyly correction was recorded, and date of dropout, if applicable).

### Outcome

Deformity correction implies complete extension of the finger, with no loss of flexion. We calculated the correction proportion using the fingers of participants who adhered to the treatment and achieved correction of their type and form of camptodactyly. The dropout rate consisted of the fingers of participants who did not adhere to treatment for the time established in this cohort.

### Statistical Analysis

The descriptive analysis used proportions for the categorical variables, and mean, standard deviation, median, minimum, maximum, and quartile values for the quantitative variables. Due to the low sample size, all comparative analyses consisted of non-parametric tests, such as the Wilcoxon-Mann-Whitney test. In addition, we presented the median and interquartile range of the six cohorts. Categorical variables were correlated in contingency tables and evaluated with the Fisher exact test.

## Results

The current study analyzed data from the medical records of 38 participants and a total of 93 affected fingers. Table 1 describes the sociodemographic characteristics. There was no association between sex and camptodactyly types according to the Fisher exact test ( *p* = 0.124).

**Table 1:** Distribution of the participants according to sex and camptodactyly type

Type		I	II	III	Total	*p* *
Sex	Male	10	1	6	17	0.124
	Female	12	6	3	21	

**Note:** *Fisher exact test.

Table 2 shows the distributions of camptodactyly types and forms in each of the six cohorts. The rigid form prevailed in our sample, affecting 60 fingers (n = 93; *0* = 0.007). Considering each camptodactyly type, the rigid and flexible forms did not present the same proportions ( *p* = 0.003) For type III, the prevalence of the rigid and flexible forms was different ( *p* < 0.001), and the rigid form occurred in 85% of the affected fingers. Considering each finger ( Table 2 ), type II presented an association between the form and the affected finger ( *p* = 0.005). The little finger demonstrates a greater prevalence of the rigid than the flexible form ( *p* = 0.027).

**Table 2:** Prevalence of types and forms of camptodactyly per affected finger

Type	Form	N	Index	Middle	Ring	Little	*p* *
I	Rigid	22	0	7	5	10	0.926
	Flexible	23	0	6	5	12	
	Total	45	0	13	10	22	
II	Rigid	9	0	0	0	9	0.005
	Flexible	5	0	2	2	1	
	Total	14	0	2	2	10	
III	Rigid	29	2	8	10	9	> 0.99
	Flexible	5	0	1	2	2	
	Total	34	2	9	12	11	

**Note:** *Fisher exact test.

Table 3 shows that the little finger was the most affected ( *p* < 0.001), representing 46% of the fingers with camptodactyly. The middle and ring fingers were equally affected (26%). The least affected finger was the index finger, representing 2% of the total. A total of 20 participants had unilateral involvement: 6 subjects had multiple fingers affected, and 14 had a single finger with camptodactyly. And 18 participants presented bilateral involvement, 10 of them with multiple fingers affected. One participant presented both forms, rigid and flexible, simultaneously.

**Table 3:** Occurrence of camptodactyly in the affected fingers

Type	N	Index: n (%)	Middle: n (%)	Ring: n (%)	Little: n (%)	*p* *
I	45	0 (0.0)	13 (28.9)	10 (22.2)	22 (48.9)	< 0.001
II	14	0 (0.0)	2 (14.3)	2 (14.3)	10 (71.4)	< 0.001
III	34	2 (5.9)	9 (26.5)	12 (35.4)	11 (32.4)	0.023
Total	93	2 (2.2)	24 (25.8)	24 (25.8)	43 (46.2)	< 0.001

**Note:** *Proportion test.

Table 4 shows the dropout frequency. From the 93 fingers included, 39 abandoned the treatment. Type II had the highest (78.6%) dropout frequency, whereas type III had the lowest (23.5%). The proportion of dropouts among participants with type-III in the flexible form was of zero. Despite this, there was no association between camptodactyly form and dropout (type I: *p* = 0.075; type II: *p* > 0.99; and type III: *p* = 0.309).

**Table 4:** Distribution of frequencies of the fingers of participants who dropped out of the treatment before achieving correction

Type	Form	N	Dropout: n (%)	*p* *
I	Rigid	22	13 (59.1)	0.075
	Flexible	23	7 (30.4)	
	Total	45	20 (44.4)	
II	Rigid	9	7 (77.8)	> 0.99
	Flexible	5	4 (80.0)	
	Total	14	11 (78.6)	
III	Rigid	29	8 (27.6)	0.309
	Flexible	5	0 (0.0)	
	Total	34	8 (23.5)	

**Note:** *Fisher exact test.

Table 5 shows the proportion of camptodactyly correction with static orthoses. Of the total of 54 fingers of participants adhering to the treatment, 38 (71.7%) presented complete correction. The correction rate was of 59% for fingers with rigid camptodactyly, and of 86% for fingers with flexible camptodactyly. There was no association between camptodactyly form and correction, but there was a trend favoring type III in the flexible form ( *p* = 0.053).

**Table 5:** Distribution of frequencies of the fingers of participants who adhered to treatment and achieved camptodactyly correction

Type	Form	N	Correction: n (%)	*p* *
I	Rigid	9	9 (100.0)	0.280
	Flexible	16	13 (81.2)	
	Total	25	22 (88.0)	
II	Rigid	2	0 (0.0)	0.333
	Flexible	1	1 (100.0)	
	Total	3	1 (33.3)	
III	Rigid	21	10 (47.6)	0.053
	Flexible	5	5 (100.0)	
	Total	26	15 (57.7)	

**Note:** *Fisher exact test.

Table 6 shows the time required for correction. Type-I camptodactyly presented the shortest median time until correction (two months). Type-III rigid camptodactyly presented the longest median time until correction (24 months). There was no association regarding the time until correction between the rigid and flexible forms in type I ( *p* = 0.705). We could not calculate this association for type II because there was no correction for the rigid form. Type III presented an association between the time until correction and the rigid or flexible forms. Correction of the flexible form occurred in sooner ( *p* = 0.006).

**Table 6:** Distribution of the time in months until correction of camptodactyly with static orthoses

Type	Form	N	Mean	SD	Min.	Max.	Median	Q1	Q3	*p* *
I	Rigid	9	7	±9	1	22	4	1	11	0.705
	Flexible	13	3	±4	1	15	2	2	4	
	Total	22	5	±6	1	22	2	1	4	
II	Rigid	0	0	±0	0	0	0	0	0	NA
	Flexible	1	7	NA	7	7	7	7	7	
	Total	1	7	NA	7	7	7	7	7	
III	Rigid	10	18	±8	9	24	24	9	24	0.006
	Flexible	5	6	±3	4	9	4	4	9	
	Total	15	14	±9	4	24	9	9	24	

**Abbreviations:** Max, maximum; Min, minimum; NA, not applicable; Q1, first quartile; Q3, third quartile; SD, standard deviation.

**Notes:** *Mann-Whitney test. The values of mean ± SD, minimum, maximum, median, Q1, and Q3 refer to the months until correçtion.

## Discussion

Consistent with the literature, 16 24 25 the little finger was the most affected in the sample of the present study. In contrast, the literature reported a bilaterality rate of 75%, 4 but this rate was lower than 50% in our patients. In a systematic review, Wang et al. 8 could not determine the best treatment for camptodactyly. However, there was consensus among the authors regarding the inaccuracy of the surgical outcome and the need to use orthoses before and after surgery.

The use of orthoses would be the way to maintain the correction achieved or even start it. Most of the articles in the literature agreed that the conservative approach with orthoses should be the first-line treatment, restricting surgery to severe cases that could not be corrected with the use of these devices. 5 8 9 14 15 17 18 24 26 27 28 29 30

The most common postoperative complications reported by Almeida et al. 16 were vascular structure injury, scar tension, and loss of full flexion of the treated finger. However, functionally, incomplete extension is better than loss of flexion. 16

The results of the current study provide evidence that keeping the finger in flexion for many years can lead to a structured deformity and possible ankylosis of the PIP joint. Therefore, ankylosis became a contraindication for conservative treatment in our service.

The dropout rate was high among participants of the present study, but the literature lacks data on dropout during conservative treatment. Participants with type-II camptodactyly presented the highest dropout rate, followed by subjects with type I (44%). Some participants with type-I camptodactyly had their first orthosis made and did not return. The dropout rate among participants with type-III camptodactyly was the lowest. We believe that, because of the severity of type-III camptodactyly, which affects several fingers simultaneously and has been associated with syndromes, 5 these participants have more reasons to remain in treatment and a higher commitment from their caregivers and legal guardians. For type II, which appears in adolescence, 5 participants may present higher rates of dropout because they are old enough to give their opinion, accept the deformity, and decide whether or not to continue with treatment. Another hypothesis for the high number of participants who abandoned treatment would be the fact that they were looking for a quick solution, such as surgery, and when they found another type of longer approach, they gave up. This data was not found in the literature, but was observed in the rehabilitation clinic for this population.

The main outcome, camptodactyly correction with static orthoses, was calculated only for the fingers of participants who adhered to the conservative treatment. Correction of the rigid form of type I was complete, and the flexible form was corrected in 81.2% of the cases. Type-II correction occurred in 33% of the cases, with the flexible form being completely corrected, as described by Goffin et al. 15 These authors reported that flexible type-II camptodactyly is more easily correctable with orthoses. For type III, the flexible form was completely corrected, while the rigid form presented a correction rate of 48%. Overall, rigid camptodactyly was corrected in 59% of the fingers, and flexible camptodactyly, in 86% of the fingers within the cohort observation period. It is worth highlighting that most subjects from our cohorts used orthoses with dorsal support, which have not been described in any study consulted. We believe that this difference facilitates deformity correction, as the dorsal support enables better positioning of the PIP joint in the orthosis.

## Conclusion

The use of static orthoses for camptodactyly treatment, especially types I and III, started early and has proven to be a safe, effective, and low-complexity solution. After analyzing the data from our sample, we consider that conservative treatment can and should be indicated as the first alternative for camptodactyly correction. However, it is necessary to make it clear to the patient/legal guardian that discipline in the use of orthoses is a decisive factor in achieving the successful correction of the deformity.
