# Supplementary material for: Conservative Treatment of Camptodactyly with use of Orthoses: A Retrospective Cohort
Source: Rev Bras Ortop (Sao Paulo). 2024 Sep 4;59(4):e564–71. doi: 10.1055/s-0044-1786199 (PMC11374391; doi:10.1055/s-0044-1786199)
Supplement: Related texts

# Research article: Tratamento conservador da camptodactilia com órteses: Coorte retrospectiva

**Keywords:** articulações dos dedos, deformidades congênitas da mão, órteses, reabilitação, terapia ocupacional

## Abstract

**Objetivo** Avaliar os resultados do tratamento conservador com o uso de órteses estáticas confeccionadas pelo Setor de Terapia Ocupacional da nossa instituição em participantes com camptodactilia dos tipos I, II e III, em suas formas rígida ou flexível, descrever os dados demográficos e clínicos, e avaliar o número de abandonos no período do tratamento.

**Métodos** O projeto foi aprovado pelo Comitê de Ética em Pesquisa em Seres Humanos, com número de protocolo CAAE 20300419.3.0000.5273. Todos os prontuários utilizados na pesquisa foram disponibilizados pela nossa instituição. Não foi feito o uso de termo de consentimento livre e esclarecido (TCLE) para este estudo retrospectivo devido à impossibilidade de contactar o elevado número de participantes. Foram incluídos os prontuários de 38 participantes atendidos no Ambulatório de Terapia Ocupacional de 2013 a 2019.

**Resultados** Dos 54 dedos tratados com órteses, 38 foram totalmente corrigidos. As taxas de correção foram as seguintes: tipo I na forma rígida –100%; tipo I na forma flexível –81,2%; tipo II na forma rígida – 0%; tipo II na forma flexível – 100%; tipo III na forma rígida –47,6%; e tipo III na forma flexível –100%. Do total de 93 dedos incluídos, 42% abandonaram o tratamento.

**Conclusão** O uso de órteses estáticas é uma alternativa segura ao procedimento cirúrgico e de baixa complexidade de execução para o tratamento da camptodactilia.

## Introdução

A camptodactilia se caracteriza por uma deformidade em flexão permanente da articulação interfalangeana proximal (IFP) de um ou vários dedos, exceto o polegar, de causa não traumática. 1 O quinto dedo é geralmente o mais acometido, e pode haver a presença de hiperextensão da articulação metacarpofalangeana (MCF). 2 3 Segundo Monteiro e Almeida, 4 a camptodactilia é uma deformidade em flexão no plano anteroposterior ( Fig. 1 ).

**Fig. 1:** Apresentação clínica de paciente com camptodactlia. Flexão da articulação interfalangeana proximal (IFP) do quinto dedo.

Segundo a classificação de Benson et al., 5 a camptodactilia se divide em: tipo I – camptodactilia infantil, que é forma mais comum, e geralmente acomete o dedo mínimo de forma isolada ( Fig. 2A ); tipo II – camptodactilia da adolescência, que é predominante no sexo feminino, e clinicamente se apresenta como a de tipo I ( Fig. 2B ); tipo III – presente desde o nascimento, acomete vários dedos, e é geralmente bilateral e associada a síndromes e outras malformações, com formas rígidas e acentuadas ( Fig. 2C ).

**Fig. 2:** Tipos de camptodactilia segundo a classificação de Benson et al. 5 ( **A** ) Tipo I: flexão da articulação IFP dos dedos médio e mínimo. ( **B** ) Tipo II: flexão da articulação IFP do dedo mínimo. ( **C** ) Tipo III: flexão da articulação IFP dos dedos médio, anelar e mínimo.

A camptodactilia é indolor, e afeta aproximadamente 1% da população. 6 Há famílias em que há um traço autossômico dominante com penetrância variável. 7 Em outros casos, a deformidade aparece de forma casual. Aproximadamente 75% dos casos são bilaterais e com progressão durante o estirão de crescimento esquelético, principalmente nas faixas etárias entre 1 e 4 e entre 10 e 14 anos. 4

Na camptodactilia, diversas estruturas se apresentam de forma anômala no dedo afetado. Várias delas foram implicadas como causa primária, mas não há consenso. 8 Ela pode se manifestar devido a alterações em várias estruturas responsáveis pelo equilíbrio articular.

Podem ser encontrados encurtamento do tendão flexor superficial do dedo, da pele, da cápsula articular da articulação IFP, da aponeurose, anomalias na inserção do músculo lumbrical, ou insuficiência na musculatura intrínseca. Todas essas estruturas foram relacionadas como causas da camptodactilia, mas nenhuma delas foi comprovada como fator etiológico 1 2 8 9 10 11 12 13 14

Goffin et al. 15 relataram que, nos pacientes que apresentam até 30° de flexão, a função da mão não é afetada, mas a partir de 60°, ela fica comprometida. O tratamento cirúrgico para correção da camptodactilia tem como complicação mais comum a perda da flexão total do dedo, e, de acordo com Almeida et al., 16 é mais desejável uma extensão incompleta do que a flexão deficiente. Além disso, mesmo em dedos tratados cirurgicamente, o uso de órteses é indicado. 17 18 19 O uso de órteses ( Figs. 3A-D , 4A-C e 5A-D ) para o tratamento da camptodactilia se dá pelo princípio da remodelação dos tecidos moles, um conceito fundamental para a teoria e técnica de imobilização conhecidas empiricamente desde os tempos antigos. Lento, leve e prolongado, o estresse faz com que os tecidos moles se remodelem ou cresçam. 20

**Fig. 3:** Modelos de órteses estáticas com apoio dorsal utilizadas para a camptodactilia do tipo I. ( **A** ) Órtese estática para camptodactilia flexível em dedo mínimo; ( **B** ) órtese estática para camptodactilia flexível em dedo médio; ( **C** ) órtese estática para camptodactilia rígida em dedo minimo com a articulação metacarpofalangeana (MCF) fletida, vista volar; e ( **D** ) órtese estática para camptodactilia rígida em dedo mínimo com a articulação MCF fletida, vista lateral.

**Fig. 4:** Modelos de órteses estáticas com apoio dorsal para a camptodactilia do tipo II rígida. ( **A** ) Camptodactilia do tipo II rígida bilateral: flexão da articulação IFP do dedo mínimo; ( **B** ) órtese estática para camptodactilia rígida em dedo minimo com a articulação MCF fletida, vista dorsal; e ( **C** ) órteses estáticas curtas para uso diurno.

**Fig. 5:** Modelos de órteses estáticas com apoio dorsal utilizadas para a camptodactilia do tipo III rígida. ( **A** ) Órtese estática para camptodactilia rígida em dedo minimo e anelar, vista lateral; ( **B** ) órteses estáticas para camptodactilia rígida em dedos mínimo e anelar da mão direita, e dedos mínimo, anelar e médio da mão esquerda; ( **C** ) órtese estática para camptodactilia englobando todos os dedos e com a articulação MCF fletida; e ( **D** ) órtese estática para camptodactilia englobando todos os dedos e com a articulação MCF fletida.

A razão primordial que direciona a escolha pelo uso de órteses é o princípio biológico de que os tecidos conjuntivos podem ser remodelados. A remodelação é uma resposta ao tempo e ao estresse que esse tecido recebe; é um rearranjo físico da matriz extracelular do tecido. Não é um processo rápido: está diretamente associado à frequência de uso e ao tempo total de uso diário. 21 Para alcançar a correção de contraturas articulares, força constante e de baixa carga deve ser aplicada por período suficiente para causar o estiramento e a criação de novo tecido. Também se fazem necessários ajustes para acomodar as alterações alcançadas e manter o alinhamento da força mecânica. Apesar das controvérsias acerca do mecanismo de ação sobre o tecido mole, o resultado é que ele se adapta à presença ou à ausência de força, e aumenta ou diminui de tamanho, se remodelando para acomodar o estresse recebido. 22 Tecidos vivos têm mecanossensores para detectar pressão e mudanças no direcionamento de forças; eles detectam o toque, a pressão e o alongamento. A chave é estabelecer a quantidade de estímulo necessário para promover a modificação e a remodelação do tecido celular. As primeiras observações da influência das forças sobre a forma e a função dos tecidos têm em torno de 150 anos. Mas ainda há muito a ser descoberto. 23

A relevância deste estudo se dá pela multiplicidade de tipos de apresentação da camptodactilia, pela multiplicidade de estruturas potencialmente envolvidas, e pela consideração de que a alternativa do tratamento cirúrgico geralmente acarreta a perda da flexão completa no dedo tratado.

O objetivo deste trabalho é avaliar o tratamento conservador com o uso de órteses estáticas em todos os tipos de camptodactilia nos pacientes atendidos no nosso serviço.

## Materiais e Métodos

Este estudo foi aprovado pelo Comitê de Ética em Pesquisa em Seres Humanos sob o número CAAE: 20300419.3.0000.5273. Todos os prontuários utilizados foram disponibilizados pela nossa instituição. Não foi feito o uso de termo de consentimento livre e esclarecido (TCLE) neste estudo retrospectivo devido à impossibilidade de contactar o elevado número de participantes.

## Desenho do Estudo

Trata-se de estudo observacional do tipo coorte retrospectivo. Foram definidas seis coortes, uma para cada tipo de camptodactilia (I, II ou III), com cada apresentação rígida ou flexível de acordo com a classificação de Benson et al.. 5 Para cada uma das seis coortes, foi avaliada a proporção de correção dos pacientes que aderiram ao tratamento e calculado o tempo médio para a correção do tipo e forma de camptodactilia. Foi verificada a frequência dos dedos acometidos. Também foi avaliada a proporção de abandono do tratamento conservador. Consideramos a correção da camptodactilia a extensão completa do dedo, sem perda da flexão.

## Composição da Amostra

Foram incluídos neste estudo os prontuários de pacientes diagnosticados pelo Centro de Atenção Especializada (CAE) da Mão com camptodactilia dos tipos I, II e III em suas formas rígida ou flexível. Foram excluídos todos os prontuários de pacientes submetidos a cirurgia por outras deformidades associadas e pacientes portadores de outro tipo de deformidade congênita não relacionada com a camptodactilia.

## Coleta de Dados

Foi utilizado o programa Epi Info (Centers for Disease Control and Prevention, Atlanta, GA, Estados Unidos), versão 7.2.3.1, e foi desenvolvido um instrumento do tipo formulário especificamente para esta pesquisa. Os dados foram coletados seguindo o protocolo criado para este estudo. Foram coletados os dados demográficos dos 38 pacientes, os dados referentes à ocorrência de camptodactilia (dedo acometido, tipo e forma de camptodactilia), além de dados sobre a correção ou o abandono, e o tempo de tratamento (data de nascimento, data de início e do final do acompanhamento, data do registro da correção da camptodactilia e data do abandono, caso houvesse).

## Desfecho

Foi considerada como correção de deformidade a extensão completa do dedo, sem a perda da flexão. A proporção de correção foi calculada com os dedos dos participantes que aderiram ao tratamento e que atingiram a correção de seu tipo e forma de camptodactilia. A proporção de abandono foi calculada apenas para os dedos dos participantes que não aderiram ao tratamento pelo tempo estabelecido nesta coorte.

## Análise Estatística

A análise descritiva foi feita com proporções para variáveis categóricas e para variáveis quantitativas, e foram utilizados valores de média e desvio padrão, mediana, mínimo, máximo e quartis. Devido ao baixo número amostral deste estudo, todas as análises comparativas foram feitas por meio de testes não paramétricos, como o teste de Wilcoxon-Mann-Whitney, e foram apresentadas a mediana e intervalo interquartil das seis coortes. Variáveis categóricas foram correlacionadas em tabelas de contingência e avaliadas pelo teste exato de Fisher.

## Resultados

Neste estudo, foram analisados os dados dos prontuários de 38 participantes com um total de 93 dedos acometidos. As características sociodemográficas estão descritas na Tabela 1 . Não houve associação entre a variável sexo e os tipos camptodactilia de acordo com o teste exato de Fisher ( *p* = 0,124).

**Tabela 1:** Distribuição dos participantes por sexo e tipo de camptodactilia

Tipo		I	II	III	Total	*p* *
Sexo	Masculino	10	1	6	17	0,124
	Feminino	12	6	3	21	

**Nota:** *Teste exato de Fisher.

A Tabela 2 mostra as distribuições de ocorrência de camptodactilia em cada uma das seis coortes. A forma rígida prevaleceu nesta amostra, e estava presente em 60 dos 93 dedos ( *p* = 0.007). Considerando cada tipo de camptodactilia, as formas rígida e flexível também não foram observadas nas mesmas proporções ( *p* = 0.003). O tipo III teve prevalências diferentes entre as formas rígida e flexível ( *p* < 0,001), e a forma rígida estava presente em 85% dos dedos acometidos. Quando consideramos as ocorrências em cada dedo ( Tabela 2 ), no tipo II há associação nas ocorrências entre a forma e o dedo acometido ( *p* = 0.00 5). O dedo mínimo parece demonstrar maior prevalência da forma rígida em relação à flexível ( *p* = 0.027).

**Tabela 2:** Prevalência dos tipos e formas de camptodactilia por dedo acometido

Tipo	Forma	N	Indicador	Médio	Anelar	Mínimo	*p* *
I	Rígida	22	0	7	5	10	0,926
	Flexível	23	0	6	5	12	
	Total	45	0	13	10	22	
II	Rígida	9	0	0	0	9	0,005
	Flexível	5	0	2	2	1	
	Total	14	0	2	2	10	
III	Rígida	29	2	8	10	9	> 0,99
	Flexível	5	0	1	2	2	
	Total	34	2	9	12	11	

**Nota:** *Teste exato de Fisher.

A Tabela 3 mostra que o dedo mínimo foi o mais acometido ( *p* < 0,001), e representou 46% dos dedos com camptodactilia; dedos médios e anelares foram acometidos em 26% dos casos cada, e o dedo menos acometido foi o indicador, com 2% do total. Ao todo, 20 participantes tiveram acometimento unilateral: 6 participantes com múltiplos dedos acometidos e 14 com apenas 1 dedo com camptodactilia. E 18 participantes tiveram acometimento bilateral, sendo 10 com múltiplos dedos acometidos. Um participante apresentou as duas formas (rígida e flexível) concomitantemente.

**Tabela 3:** Ocorrência de camptodactilia nos dedos acometidos

Tipo	N	Indicador: n (%)	Médio: n (%)	Anelar: n (%)	Mínimo: n (%)	*p* *
I	45	0 (0,0)	13 (28,9)	10 (22,2)	22 (48,9)	< 0,001
II	14	0 (0,0)	2 (14,3)	2 (14,3)	10 (71,4)	< 0,001
III	34	2 (5,9)	9 (26,5)	12 (35,4)	11 (32,4)	0.023
Total	93	2 (2,2)	24 (25,8)	24 (25,8)	43 (46,2)	< 0,001

**Nota:** *Teste de proporção.

A Tabela 4 mostra a frequência de abandono: dos 93 dedos dos participantes, 39 abandonaram o tratamento. O tipo II apresentou a maior frequência de abandono (78,6%), e o tipo III, a menor (23,5%). A proporção de abandono entre os participantes com a forma flexível foi de zero para o tipo III. Apesar disso, não houve associação entre a forma de camptodactilia e o abandono em nenhum tipo (tipo I: *p* = 0.075; tipo II: *p* > 0.99; e tipo III: *p* = 0.309).

**Tabela 4:** Distribuição de frequências dos dedos de participantes que abandonaram o tratamento antes de alcançar a correção

Tipo	Forma	N	Abandono: *n* (%)	*p* *
I	Rígida	22	13 (59,1)	0,075
	Flexível	23	7 (30,4)	
	Total	45	20 (44,4)	
II	Rígida	9	7 (77,8)	> 0,99
	Flexível	5	4 (80,0)	
	Total	14	11 (78,6)	
III	Rígida	29	8 (27,6)	0,309
	Flexível	5	0 (0,0)	
	Total	34	8 (23,5)	

**Nota:** *Teste exato de Fisher.

A Tabela 5 mostra a proporção de correção da camptodactilia com o uso de órteses estáticas: Do total de 54 dedos de participantes que aderiram ao tratamento, 38 (71,7%) foram totalmente corrigidos. A camptodactilia rígida foi corrigida em 59% dos dedos, e a flexível, em 86%. Não houve associação entre a forma da camptodactilia e a correção, mas houve uma tendência no tipo III flexível ( *p* = 0.053).

**Tabela 5:** Distribuição de frequências de dedos dos participantes que aderiram ao tratamento e alcançaram a correção da camptodactilia

Tipo	Forma	N	Correção: *n* (%)	*p* *
I	Rígida	9	9 (100,0)	0,280
	Flexível	16	13 (81,2)	
	Total	25	22 (88,0)	
II	Rígida	2	0 (0,0)	0,333
	Flexível	1	1 (100,0)	
	Total	3	1 (33,3)	
III	Rígida	21	10 (47,6)	0,053
	Flexível	5	5 (100,0)	
	Total	26	15 (57,7)	

**Nota:** *Teste exato de Fisher.

Na Tabela 6 estão os tempos necessários até a correção. A camptodactilia do tipo I foi a que levou O menor tempo mediano até a correção (dois meses). A camptodactilia do tipo III rígida teve o maior tempo mediano observado até a correção (24 meses). Não houve associação relativa ao tempo decorrido até a correção e as formas rígida e flexível no tipo I ( *p* = 0,705), e no tipo II não foi possível calcular, porque não tivemos nenhuma correção na forma rígida, mas, no tipo III, houve associação entre o tempo decorrido até a correção e as formas rígida e flexível: a forma flexível foi corrigida em menor tempo ( *p* = 0,006).

**Tabela 6:** Distribuição do tempo, em meses, decorrido até a correção da camptodactilia com o uso de órteses estáticas

Tipo	Forma	N	Média	DP	Mín.	Máx.	Mediana	Q1	Q3	*p*
I	Rígida	9	7	±9	1	22	4	1	11	0,705
	Flexível	13	3	±4	1	15	2	2	4	
	Total	22	5	±6	1	22	2	1	4	
II	Rígida	0	0	±0	0	0	0	0	0	NA
	Flexível	1	7	NA	7	7	7	7	7	
	Total	1	7	NA	7	7	7	7	7	
III	Rígida	10	18	±8	9	24	24	9	24	0,006
	Flexível	5	6	±3	4	9	4	4	9	
	Total	15	14	±9	4	24	9	9	24	

Abreviaturas: DP, desvio padrão; Máx., máximo; Mín., mínimo; NA, não se aplica; Q1, primeiro quartil; Q3, terceiro quartil.

**Notas:** *Teste de Mann-Whitney. Os valores de média ± DP, mínimo, máximo, mediana, Q1 e Q3 se referem aos meses decorridos até a correção.

## Discussão

Assim como na literatura, 16 24 25 na amostra deste estudo, o dedo mínimo foi o mais afetado; mas, em relação à bilateralidade, que nos estudos consultados gira em torno de 75%, 4 na nossa amostra não chegou a 50% dos participantes. Na revisão sistemática feita por Wang et al., 8 não foi possível direcionar o melhor tratamento para a camptodactilia; entretanto, houve consenso entre os autores sobre a imprecisão do resultado cirúrgico e sobre a necessidade do uso de órteses como pré ou pós-intervenção.

O uso de órteses seria a forma de manter a correção alcançada ou até mesmo de iniciá-la. Em sua maioria, os artigos na literatura concordam que a abordagem conservadora com o uso de órteses deva ser a primeira escolha de tratamento, ficando a cirurgia restrita a casos muito graves que não tenham sido corrigidos com o uso das órteses. 5 8 9 14 15 17 18 24 26 27 28 29 30

As complicações mais comuns no pós-operatório relatadas por Almeida et al. 16 são: lesão de estruturas vasculares, tensão cicatricial e perda da flexão total do dedo tratado. Porém, funcionalmente, uma extensão incompleta é melhor do que a perda da flexão. 16

Os resultados deste estudo fornecem indícios de que a permanência do dedo em flexão por muitos anos pode levar a uma deformidade estruturada e possível anquilose da articulação IFP. Desta forma, a presença de anquilose se tornou uma contraindicação para o tratamento conservador no nosso serviço.

Houve um alto índice de abandono entre os participantes deste estudo; porém, na literatura consultada, não foi encontrado nenhum dado em relação ao abandono durante o tratamento conservador. Os participantes com camptodactilia do tipo II foram os que mais abandonaram (seguidos dos participantes do tipo I, com 44%). Alguns participantes com camptodactilia do tipo I compareceram para a confecção de uma primeira órtese e não retornaram. A proporção de abandono entre os participantes com camptodactilia do tipo III foi a menor de todas. Acreditamos que, devido à gravidade da camptodactilia do tipo III, que acomete diversos dedos simultaneamente e está ligada a síndromes, 5 esses participantes tenham mais motivos para permanecer em tratamento e tenham maior comprometimento por parte de seus cuidadores e responsáveis. Já para o tipo II, que tem seu surgimento na adolescência, 5 os participantes podem apresentar maiores proporções de abandono pelo fato de terem idade para opinar, aceitar a deformidade e decidir dar ou não seguimento ao tratamento. Outra hipótese para o elevado número de participantes que abandonaram o tratamento seria o fato de eles buscarem uma solução rápida, como uma cirurgia, e, ao encontrarem outro tipo de abordagem mais demorada, desistem. Esse dado não foi encontrado na literatura; é fruto da observação na clínica na área da reabilitação com essa população.

O desfecho principal, a correção da camptodactilia com o uso de órteses estáticas, foi calculado apenas com os dedos dos participantes que aderiram ao tratamento conservador. O tipo I da forma rígida foi totalmente corrigido, e a forma flexível foi corrigida em 81,2% dos casos. O tipo II foi corrigido em 33% dos casos, sendo a forma flexível totalmente corrigida, assim como descrito por Goffin et al., 15 que relatam que a camptodactilia do tipo II flexível é mais facilmente corrigível com uso de órteses. Para o tipo III, a forma flexível foi totalmente corrigida, e a rígida teve a proporção de 48% de correção. De forma global, a camptodactilia rígida foi corrigida em 59% dos dedos, e a flexível, em 86% dos dedos dentro do período de observação das coortes. É importante ressaltar que, na maioria da população deste estudo, foram utilizadas órteses com apoio dorsal, o que não está descrito em nenhum dos estudos consultados. Acreditamos que esse diferencial tenha sido um facilitador para o alcance da correção da deformidade, visto que o apoio dorsal permite um melhor posicionamento da articulação IFP na órtese.

## Conclusão

O uso de órteses estáticas para o tratamento da camptodactilia, principalmente a dos tipos I e III, iniciado precocemente, se mostrou como uma solução segura, eficaz e de baixa complexidade de execução. Após a análise dos dados desta amostra, consideramos que o tratamento conservador pode e deve ser indicado como primeira alternativa para a correção da camptodactilia; porém, é necessário deixar claro ao paciente/responsável que a disciplina no uso das órteses é fator decisivo para o alcance da correção da deformidade.
